# COVID-19 and the increased risk of myopia and digital eye strain

**DOI:** 10.31744/einstein_journal/2021CE6491

**Published:** 2021-05-13

**Authors:** Lorenzo Ferro Desideri, Marcos Roberto Tovani-Palone

**Affiliations:** 1 Università di Genova GenovaGE Italy Università di Genova, Genova, GE, Italy.; 2 Universidade de São Paulo Faculdade de Medicina de Ribeirão Preto Ribeirão PretoSP Brazil Faculdade de Medicina de Ribeirão Preto, Universidade de São Paulo, Ribeirão Preto, SP, Brazil.

Dear Editor,

The current pandemic of coronavirus disease 2019 (COVID-19), caused by severe acute respiratory syndrome coronavirus 2 (SARS-CoV-2), has had catastrophic effects on human society in several aspects, including social, economic, and public health.^(^^1^^,^^2^^)^ Severe cases of the disease are often associated with respiratory, renal, and cardiovascular complications, as well as with other related risks.^(^^3^^,^^4^^)^

More specifically, the COVID-19 pandemic has considerably modified our lifestyle due to mainly “social distancing” measures that has been adopted to reduce the SARS-CoV-2 infection rates, protect the vulnerable populations, and prevent the collapse of health systems.^(^^5^^)^ For these reasons, several changes in our daily routine have occurred, including staring for long periods at computer monitors and television screens.^(^^6^^)^

Currently, ophthalmologists all over the world are alarmed by the risk of an increased incidence of myopia progression, also called “quarantine myopia”, and digital eye strain as a result of the current restriction measures that have led to an imposed home confinement.^(^^7^^)^ In fact, a long period of time home is more likely related to longer periods in front of tablets, smartphones, and computer monitors. In this regard, the use of handheld digital devices has been proven to be associated with alterations in the accommodation process (increased lag and concomitant reduced amplitude) and reduced fusional convergence, causing digital eye strain.^(^^8^^)^ Moreover, handheld digital devices and computer monitors may reduce the blink rate and affect the tear stability, with the risk of developing dry eye syndrome.^(^^9^^)^

In addition to this, it is widely recognized the negative role played by intense near-work activity (such as reading, studying, watching TV, or playing video games etc.) and a decreased time spent outdoors towards the progression of myopia, probably due to dopaminergic pathways influenced by these environmental factors.^(^^10^^)^ It is already known that an increased prevalence of myopia can lead to a higher prevalence of myopia-induced sight-threatening complications, including retinal detachment, retinal tears, and neovascularization in pathological myopias.^(^^11^^)^

Given the current situation, managers and health authorities should consider of paramount importance the expanding of health services to ophthalmological monitoring of the population,^(^^12^^)^ in order to prevent the increased prevalence of non-negligible ocular diseases, including myopia and ocular surface disorders (such as digital eye strain). Moreover, educational campaigns on this subject matter should also be implemented as soon as possible, in this case, with a focus on the importance of avoiding spending much time on computer monitors and handheld digital devices in order to preserve ocular health.
