# Supplementary material for: COVID-19 and the increased risk of myopia and digital eye strain
Source: Einstein (Sao Paulo). 2021 May 13;19:eCE6491. doi: 10.31744/einstein_journal/2021CE6491 (PMC8121374; doi:10.31744/einstein_journal/2021CE6491)
Supplement: Translation

# Translation: COVID-19 e o risco aumentado de miopia e fadiga ocular digital

**Authors:** Lorenzo Ferro Desideri, Marcos Roberto Tovani-Palone

**Affiliations:** 1 GenovaGE Itália Università di Genova, Genova, GE, Itália.; 2 Ribeirão PretoSP Brasil Faculdade de Medicina de Ribeirão Preto, Universidade de São Paulo, Ribeirão Preto, SP, Brasil.

Caro Editor,

A atual pandemia da doença pelo coronavírus 2019 (COVID-19), causada pelo coronavírus da síndrome respiratória aguda grave 2 (SARS-CoV-2), tem apresentado efeitos catastróficos para a sociedade em diversos níveis, incluindo problemas sociais, econômicos e de saúde pública.^(^^1^^,^^2^^)^ Os casos graves da doença estão geralmente associados a sintomas respiratórios, renais e complicações cardiovasculares, assim como a outros riscos relacionados.^(^^3^^,^^4^^)^

Mais especificamente, a pandemia da COVID-19 tem modificado significativamente nosso estilo de vida devido, em grande parte, às medidas de “distanciamento social“ que têm sido adotadas a fim de se reduzir as taxas de infecção por SARS-CoV-2, proteger as populações vulneráveis e prevenir o colapso dos sistemas de saúde.^(^^5^^)^ Diante disso, diversas mudanças em nosso rotina diária têm ocorrido, como o aumento do tempo de permanência na frente de monitores de computadores e telas de televisores.^(^^6^^)^

Atualmente, oftalmologistas de todo o mundo estão preocupados com o risco de aumento na incidência de casos de progressão de miopia, os quais têm sido denominados de “miopia da quarentena”, além ainda dos números sobre a ocorrência de fadiga ocular digital. Ambas as condições estão relacionadas com as medidas de restrição impostas, que, por sua vez, têm levado a um confinamento doméstico compulsório.^(^^7^^)^ De fato, permanecer por mais tempo em casa está provavelmente mais associado a passar mais horas na frente de *tablets, smartphones* e monitores de computadores.

Nesse contexto, o uso de dispositivos digitais portáteis tem sido associado com alterações no processo de acomodação (aumento do LAG acomodativo e amplitude reduzida), bem como com convergência fusional diminuída, resultando em fadiga ocular digital.^(^^8^^)^ Os dispositivos digitais portáteis e monitores de computadores podem reduzir a taxa de piscadas e alterar a estabilidade lacrimal, com risco de desenvolvimento da síndrome do olho seco.^(^^9^^)^ Outra questão está relacionada ao impacto negativo da atividade intensa de trabalho de perto (como ler, estudar, assistir à televisão ou jogar videogame etc.) e da redução do tempo gasto em atividades ao ar livre para a progressão da miopia, provavelmente devido à influência desses fatores ambientais sobre as vias dopaminérgicas.^(^^10^^)^

O aumento da prevalência de miopia pode levar a uma maior frequência de complicações da visão decorrentes do próprio distúrbio ocular, incluindo o descolamento e as rupturas de retina bem como a neovascularização associada à miopia patológica.^(^^11^^)^

Dado o cenário atual, é de suma importância para gestores e autoridades de saúde proverem a expansão de serviços de saúde voltados para o acompanhamento oftalmológico da população,^(^^12^^)^ a fim de prevenir o aumento da prevalência de doenças oculares não negligenciáveis, incluindo a miopia e os distúrbios da superfície ocular (tal como a fadiga ocular digital). Além disso, campanhas educativas sobre esse assunto devem ser implementadas o mais brevemente possível, com ênfase na importância de evitar a exposição por longos períodos em frente a monitores de computadores e dispositivos digitais por­táteis, com o objetivo de preservar a saúde ocular.
